# CVDHD: a cardiovascular disease herbal database for drug discovery and network pharmacology

**DOI:** 10.1186/1758-2946-5-51

**Published:** 2013-12-18

**Authors:** Jiangyong Gu, Yuanshen Gui, Lirong Chen, Gu Yuan, Xiaojie Xu

**Affiliations:** 1 Beijing National Laboratory for Molecular Sciences, State Key Lab of Rare Earth Material Chemistry and Applications, College of Chemistry and Molecular Engineering, Peking University, Room A817, No.202, Chengfu Road, Beijing, Haidian District 100871, P. R. China

**Keywords:** Cardiovascular disease, Drug discovery, Network pharmacology, Molecular docking, Virtual screening, Herbal formula, Natural products, Medicinal herbs, Traditional Chinese medicine

## Abstract

**Background:**

Cardiovascular disease (CVD) is the leading cause of death and associates with multiple risk factors. Herb medicines have been used to treat CVD long ago in china and several natural products or derivatives (e.g., aspirin and reserpine) are most common drugs all over the world. The objective of this work was to construct a systematic database for drug discovery based on natural products separated from CVD-related medicinal herbs and to research on action mechanism of herb medicines.

**Description:**

The cardiovascular disease herbal database (CVDHD) was designed to be a comprehensive resource for virtual screening and drug discovery from natural products isolated from medicinal herbs for cardiovascular-related diseases. CVDHD comprises 35230 distinct molecules and their identification information (chemical name, CAS registry number, molecular formula, molecular weight, international chemical identifier (InChI) and SMILES), calculated molecular properties (AlogP, number of hydrogen bond acceptor and donors, etc.), docking results between all molecules and 2395 target proteins, cardiovascular-related diseases, pathways and clinical biomarkers. All 3D structures were optimized in the MMFF94 force field and can be freely accessed.

**Conclusions:**

CVDHD integrated medicinal herbs, natural products, CVD-related target proteins, docking results, diseases and clinical biomarkers. By using the methods of virtual screening and network pharmacology, CVDHD will provide a platform to streamline drug/lead discovery from natural products and explore the action mechanism of medicinal herbs. CVDHD is freely available at http://pkuxxj.pku.edu.cn/CVDHD.

## Background

Natural products have been an important source for drug/lead discovery [1,2]. More than half of FDA-approved drugs are natural products or derivatives [3]. The most important approach in current drug discovery is through screening. However, traditional combinatorial chemistry libraries generally show limited structural diversity [4]. Through the natural selection process, natural products have vast diversities both in chemical space and pharmacological space [5-7]. High-throughput screening (HTS) *in silico* is used to reduce the number of chemicals to be tested in vitro and vivo. HTS based on molecular docking is an efficient approach to identify chemicals that could fit into the active site of target proteins. Therefore the virtual screening based on natural product database is a promising approach for drug discovery, especially for complex diseases such as cardiovascular disease.

Cardiovascular disease is regarded to be the main cause of death worldwide [8]. As a complex disease, CVD is the consequence of multiple pathogenic factors and reflects the altered interactions of many interconnected genes and gene products [9]. How to effectively and efficiently reverse these inappropriate interactions in a sick state is a critical problem. However, most drugs for CVD were designed to target a specific target and cannot be very effective [10-12]. In contrast, drugs which target multiple targets will have maximal efficacy and minimal adverse effects [13]. Polypharmacology and network pharmacology are useful approaches for understanding the mechanism and evaluating the efficacy of drugs at systems level and could aid in design and development of drugs with higher success rate [14-18]. Meanwhile, biological pathways of diseases or biological process are important networks and usually associate with clinical biomarkers [19]. By investigating the effects of compounds on the biological pathway network, researchers can easily evaluate the potency of compounds and thus move basic biological discoveries into the clinic applications.

The herbal medicines which contain biologically active natural products for CVD have been used for thousands of years in China. However, the ingredients are too complex (usually hundreds of compounds) and the biological mechanisms of herbs are not yet understood clearly [14,16,18,20]. Along with the progress of network pharmacology and the explosive growth of biomedical data, the analysis of action mechanisms of medicinal herbs at the systems level becomes possible [5,15,21-23]. Therefore, we constructed the cardiovascular disease herbal database (CVDHD) that was implemented based on natural products-target proteins interactions and integration of multi-level data to promote the drug discovery from natural products and explore the molecular basis of polypharmacology of medicinal herbs for CVD. CVDHD will also provide an efficient platform for research on traditional Chinese medicine (TCM) and translational research in complementary and alternative medicine.

## Construction and content

**Figure 1:**
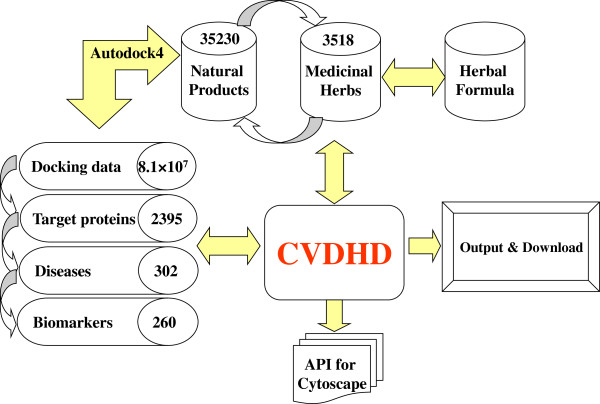
Database schema and search flow chart.

CVDHD comprises six data entities covering medicinal herbs, natural products, target proteins, docking results between all molecules and target proteins, diseases and clinical biomarkers (Figure 1). The medicinal herbs (Chinese name, Latin name and pharmacological effects) for cardiovascular-related diseases were collected from Chinese Herbalism [24] and Chinese Pharmacopoeia [25]. The structures and identification information (chemical name, CAS registry number, molecular formula, molecular weight and information of references) of natural products contained in the medicinal herbs were retrieved from CHDD [26] and UNPD [5] which were both developed by our lab in recent years. The absolute configuration of each molecule was generated by Open Babel [27] and the duplicates were deleted according to InChIKey. The molecular properties (AlogP, number of hydrogen bond acceptor and donors, etc.) were calculated by Discovery Studio.

There were two main groups of target proteins in CVDHD. One was the drug targets retrieved from DrugBank [28], another was the other proteins of human. Each protein had X-ray or NMR ligand-protein complex structures in RCSB protein data bank (http://www.rcsb.org/pdb/home/home.do). These structures were downloaded and treated to be suitable for molecular docking by Autodock4 [29] according to the procedure described previously [5]. For each protein, the binding site was defined as a 40 × 40 × 40 Å cube centered on the occupied space of the original ligand with a spacing of 0.375 Å between the grid points. The parameters of autodock4 were listed in Additional file 1. The docking score of autodock4 was chosen to evaluate the binding affinity according to binding free energy.

The information of diseases and clinical biomarkers related to CVD was collected from KEGG [30], TTD [31] and literatures manually. Finally, the data was stored in a MySQL (5.0.45) database. CVDHD was implemented as a PHP-based web application which was deployed to an Apache Tomcat server (PHP 5.1.6 and HTTPD 2.2.3). CVDHD can be accessed via the internet. Moreover, the API for Cytoscape was reserved to meet the future demands. Therefore, CVDHD can be applied seamlessly to the network pharmacology analysis by using the network analysis software such as Cytoscape and CentiBin.

## Utility and discussion

### Database interface

CVDHD had a powerful and intuitive web interface. The web pages were divided into five sections (Home, Introduction, Browse, Search and Download). A concise retrieval system for natural products was available on the homepage. User can search exactly by its identification information or get a group of compounds by the Latin name of medicinal herb. The introduction page described the database design and main functions. The browse pages contained five lists of medicinal herbs, molecules, proteins, diseases and biomarkers. The various data retrievals of drug discovery and network pharmacology can be achieved on search pages. The download page contained several links for download.

### Lead compound discovery

The lead generation is a crucial step in drug discovery [32]. Nowadays, HTS has become the major paradigm for lead discovery from chemical libraries [32,33]. Lipinski’s “rule of five” (molecular weight less than 500 Da, hydrogen bond acceptors less than 10, hydrogen bond donors less than 5, octanol-water partition coefficient less than five) [34] was a simple but powerful rule to estimate the possibility of converting a compound into a drug. CVDHD can easily screen out the molecules which obey “rule of five” or other customized thresholds. For example, user can retrieve the potentially bioactive compounds from *Panax ginseng* (one of the most important medicinal herbs used as a tonic for restoration [35]) after entering the Latin name of this herb and thresholds of molecular properties on the advanced search webpage. The output of this query will be a table containing the molecular information and download link of each compound.

Moreover, if the target proteins of a disease were clear, the receptor-based drug design will accelerate the progress of drug discovery [36]. The HTS results between all molecules and all target proteins were stored in CVDHD and user can access data by simply typing four parameters: the Uniprot accession number of the target protein, the thresholds of docking score (p*Ki*), the top percentage of rank of docking score and the logic value of whether the docking score of compounds was higher than that of original ligand in the X-ray or NMR structure of the target protein (0 or 1). It may save a lot of time for users in the process of virtual screening.

### Network pharmacology research

Complex diseases such as CVD and diabetes are caused by a variety of genetic and environmental factors [37]. The modern drugs (magic bullets) which act on single target cannot treat complex diseases effectively [38-41]. Therefore, Hopkins AL [15,22] proposed network pharmacology to explore the action mechanism of drugs in the context of biological networks such as biological pathway, gene regulatory network, protein-protein interaction network, drug-target network, etc. The drug-target network (DTN) and its derivatives (drug-drug network (DDN) and target-target network (TTN)) could have important implications in understanding the mechanism and harnessing the vast amount of data from HTS [42-45].

The drug-target network was constructed according to the various binding data between molecules and target proteins. However, there were only a small portion (less than 2%) of natural products which binding data has been reported [5]. Thus, CVDHD used molecular docking to calculate the binding affinity between all molecules and 2395 target proteins. Based on CVDHD, comprehensive research on the mechanism of the medicinal herbs ranging from the level of herbal formula to protein-compound interaction can be achieved. Moreover, user can retrieve all target proteins relating to a pathway of a disease or biological process and finally find out the potential interactions between molecules and proteins to construct DTN, DDN and TTN to unveil the mechanism of TCM. CVDHD may bridge the gap between TCM and modern drugs based on system-level analysis.

### Case study: platelet aggregation pathway based lead discovery

Platelet aggregation plays an important role in arterial thrombosis in coronary heart disease [46]. How to regulate the platelet aggregation of CVD patients is one of the keys of successful treatment. The pathway of platelet aggregation comprised nineteen target proteins [19]. If a compound can block one of these proteins, the platelet aggregation will be inhibited to some extent. That is, the more inhibition of targets, the more effective the compound would be [19,47].

We retrieved the potentially active compounds from CVDHD with the following parameters: the thresholds of docking score, the top percentage of rank of docking score were set to 7.0 and 0.10, respectively. Meanwhile, the docking score of compounds should be higher than that of original ligand. The drug-target network (Additional file 2: Table S1) was constructed by linking the compound and target protein if the docking score exceeded the threshold values. Cytoscape 2.8.3 [48] was adopted to draw the DTN and calculate the degree and betweenness centrality of each node (compound or target). The larger degree and the higher betweenness, the more effect on the inhibition of platelet aggregation of that compound will be according to the network theory. Table 1 listed the top rank of potential compounds which may be the lead compounds for inhibition of platelet aggregation for further study. However, it is a simple model to use degree centrality to evaluate the efficacy of natural products. Users can use other models to predict the efficacy in the context of drug-target network.

**Table 1:** Top potential compounds for inhibition of platelet aggregation according to degree centrality of drug-target network

**Rank**	**Compound**	**Degree**	**CAS NO.**	**Chemical name**
1	CVDHD016293	6	62218-13-7	(+)-α-viniferin
2	CVDHD027110	6	N/A	hypericinate
3	CVDHD007020	6	54352-30-6	tricrotonyltetramine
4	CVDHD012997	6	N/A	blumeanine
5	CVDHD001236	5	N/A	kadlongilactone B
6	CVDHD012285	5	225662-66-8	hypericin radical cation
7	CVDHD002703	5	50838-55-6	trisjuglone
8	CVDHD032784	5	55954-61-5	pseudohypericine
9	CVDHD029497	5	7034-04-0	tricrotonyltetramin
10	CVDHD002390	4	N/A	20(R)-21,24-Cyclo-3beta,25-dihydroxyldammar-23(24)-en-21-one
11	CVDHD014854	4	N/A	longipedlactone F
12	CVDHD034764	4	N/A	celastroline B alpha
13	CVDHD010515	4	123522-98-5	isowithametelin
14	CVDHD014739	4	N/A	eremodimer B
15	CVDHD025068	4	N/A	bismorphine B
16	CVDHD017384	4	1253379-22-4	dievodiamine
17	CVDHD021374	4	N/A	lycochinine B
18	CVDHD025464	4	511-98-8	solasodanol
19	CVDHD019403	4	129748-10-3	incarvillateine
20	CVDHD019431	4	548-04-9	hypericin
21	CVDHD029988	4	129225-31-6	aminopropylcanavalmine
22	CVDHD009071	4	77646-14-1	3β,17α-Cinchophylline
23	CVDHD015834	4	N/A	bismorphine A
24	CVDHD011497	4	N/A	dihydro-18,19 3β,17β cinchophylline

## Conclusions

CVDHD is aimed to integrate medicinal herbs, natural products, CVD-related target proteins, docking results, diseases and clinical biomarkers to be a comprehensive database for drug discovery from natural products isolated from medicinal herbs. First, CVDHD is a chemical library of natural products and ready for virtual screening. All 3D structures of natural products and information of binding site of target proteins can be accessed on the website. Second, drug/lead discovery for single target or a group of proteins related to a disease or biological process can be achieved. Finally, it is also a research platform for network pharmacology of medicinal herbs and TCM. The molecule and herb search can be applied to get compounds contained in herbs. Moreover, CVDHD is useful for study of network pharmacology of CVD-related natural products. The herbs contain a variety of ingredients and the compounds would interact with multiple cellular targets. By identifying the associations between bioactive compounds and cellular target proteins, CVDHD may bridge the gap between the systems level (herbal formula or medicinal herb) and molecular biology (compounds and proteins).

## Availability and requirements

CVDHD is freely available at http://pkuxxj.pku.edu.cn/CVDHD and there are no restrictions for academic use. The database will be updated annually.

## Abbreviations

CVD: Cardiovascular disease; NP: Natural product; DTN: Drug-target network; DDN: Drug-drug network; TTN: Target-target network; HTS: High-throughput screening; TCM: Traditional Chinese medicine.

## Competing interests

The authors declare that they have no competing interests.

## Authors’ contributions

XJX and LRC conceived the study. JYG and YSG constructed the database, performed computational analyses and interpreted the results with the help of GY. JYG designed the website and wrote the manuscript. All authors read and approved the final manuscript.

## Supplementary Material

Additional file 1 Autodock4 parameters. Click here for file

Additional file 2: Table S1 Drug-target network of potential lead compounds and target proteins of platelet aggregation pathway. Click here for file
