# Supplementary material for: CVDHD: a cardiovascular disease herbal database for drug discovery and network pharmacology
Source: J Cheminform. 2013 Dec 18;5:51. doi: 10.1186/1758-2946-5-51 (PMC3878363; doi:10.1186/1758-2946-5-51)
Supplement: Additional file 1 — Autodock4 parameters. [file 1758-2946-5-51-S1.docx]

**CVDHD: a cardiovascular disease herbal database for drug discovery and network pharmacology**

Jiangyong Gu^1^ ,Yuanshen Gui^1^, Lirong Chen^1,*^, Gu Yuan^1^, Xiaojie Xu^1,*^

^1^Beijing National Laboratory for Molecular Sciences, State Key Lab of Rare Earth Material Chemistry and Applications, College of Chemistry and Molecular Engineering, Peking University, Beijing 100871, P. R. China.

^*^ Correspondence should be addressed to Xiaojie Xu, [xiaojxu@pku.edu.cn](mailto:xiaojxu@pku.edu.cn) and Lirong Chen, [lirongc@pku.edu.cn](mailto:lirongc@pku.edu.cn).

The Additional file 1 listed the parameters used in the virtual screening by autodock4.0.

**Additional file 1: the parameters used in the virtual screening by autodock4**

outlev 1 # diagnostic output level

ligand_types H HD HS C A N NA NS OA OS F P SA S Cl CL Br BR I # ligand atom types

fld .maps.fld # grid_data_file

map .H.map # atom-specific affinity map

map .HD.map # atom-specific affinity map

map .HS.map # atom-specific affinity map

map .C.map # atom-specific affinity map

map .A.map # atom-specific affinity map

map .N.map # atom-specific affinity map

map .NA.map # atom-specific affinity map

map .NS.map # atom-specific affinity map

map .OA.map # atom-specific affinity map

map .OS.map # atom-specific affinity map

map .F.map # atom-specific affinity map

map .P.map # atom-specific affinity map

map .SA.map # atom-specific affinity map

map .S.map # atom-specific affinity map

map .Cl.map # atom-specific affinity map

map .CL.map # atom-specific affinity map

map .Br.map # atom-specific affinity map

map .BR.map # atom-specific affinity map

map .I.map # atom-specific affinity map

elecmap .e.map # electrostatics map

desolvmap .d.map # desolvation map

tstep 2.0 # translation step/A

qstep 50.0 # quaternion step/deg

dstep 50.0 # torsion step/deg

torsdof 5 0.274000 # torsional degrees of freedom and coefficient

rmstol 2.0 # cluster_tolerance/A

extnrg 1000.0 # external grid energy

e0max 0.0 10000 # max initial energy; max number of retries

ga_pop_size 150 # number of individuals in population

ga_num_evals 1000000 # maximum number of energy evaluations

ga_num_generations 27000 # maximum number of generations

ga_elitism 1 # number of top individuals to survive to next generation

ga_mutation_rate 0.02 # rate of gene mutation

ga_crossover_rate 0.8 # rate of crossover

ga_window_size 10 #

ga_cauchy_alpha 0.0 # Alpha parameter of Cauchy distribution

ga_cauchy_beta 1.0 # Beta parameter Cauchy distribution

set_ga # set the above parameters for GA or LGA

sw_max_its 300 # iterations of Solis & Wets local search

sw_max_succ 4 # consecutive successes before changing rho

sw_max_fail 4 # consecutive failures before changing rho

sw_rho 1.0 # size of local search space to sample

sw_lb_rho 0.01 # lower bound on rho

ls_search_freq 0.06 # probability of performing local search on individual

seed 1 1 # for random number generator

move $LIGAND_PDBQ_NAME$ # small molecule file

tran0 random # initial coordinates/A or "random"

quat0 random # initial quaternion or "random"

dihe0 random # initial torsions

set_ga # set the above parameters for GA or LGA

set_sw1 # set the above pseudo-Solis & Wets parameters

ga_run 1 # do this many GA or LGA runs

#analysis # do cluster analysis on results

npts GRID_DIMENSION_NPT # num.grid points in xyz

gridfld $protein_name$.maps.fld # grid_data_file

spacing GRID_SPACING # spacing(A)

receptor_types H HD HS C A N NA NS OA OS F Mg MG P SA S Cl CL Ca CA Mn MN Fe FE Zn ZN Br BR I # receptor atom types

ligand_types H HD HS C A N NA NS OA OS F P SA S Cl CL Br BR I # ligand atom types

receptor PROTEIN_PDBQT # macromolecule

gridcenter GRID_CENTER # xyz-coordinates or auto

smooth 0.5 # store minimum energy w/in rad(A)

map $protein_name$.H.map # atom-specific affinity map

map $protein_name$.HD.map # atom-specific affinity map

map $protein_name$.HS.map # atom-specific affinity map

map $protein_name$.C.map # atom-specific affinity map

map $protein_name$.A.map # atom-specific affinity map

map $protein_name$.N.map # atom-specific affinity map

map $protein_name$.NA.map # atom-specific affinity map

map $protein_name$.NS.map # atom-specific affinity map

map $protein_name$.OA.map # atom-specific affinity map

map $protein_name$.OS.map # atom-specific affinity map

map $protein_name$.F.map # atom-specific affinity map

map $protein_name$.P.map # atom-specific affinity map

map $protein_name$.SA.map # atom-specific affinity map

map $protein_name$.S.map # atom-specific affinity map

map $protein_name$.Cl.map # atom-specific affinity map

map $protein_name$.CL.map # atom-specific affinity map

map $protein_name$.Br.map # atom-specific affinity map

map $protein_name$.BR.map # atom-specific affinity map

map $protein_name$.I.map # atom-specific affinity map

elecmap $protein_name$.e.map # electrostatic potential map

dsolvmap $protein_name$.d.map # desolvation potential map

dielectric -0.1465 # <0, AD4 distance-dep.diel;>0, constant
